# Baseline Inventory of Benthic Macrofauna in German Marine Protected Areas (2020–2022) before Closure for Bottom-Contact Fishing

**DOI:** 10.3390/biology13060389

**Published:** 2024-05-28

**Authors:** Mayya Gogina, Sarah Joy Hahn, Ramona Ohde, Angelika Brandt, Stefan Forster, Ingrid Kröncke, Martin Powilleit, Katharina Romoth, Moritz Sonnewald, Michael L. Zettler

**Affiliations:** 1 Leibniz Institute for Baltic Sea Research Warnemünde, Seestrasse 15, D-18119 Rostock, Germany; katharina.romoth@io-warnemuende.de (K.R.); michael.zettler@io-warnemuende.de (M.L.Z.); 2 Senckenberg Research Institute and Museum of Nature, D-60325 Frankfurt am Main, Germany; sarah.joy.hahn@gmx.de (S.J.H.); angelika.brandt@senckenberg.de (A.B.); moritz.sonnewald@senckenberg.de (M.S.); 3 Senckenberg am Meer, Department for Marine Research, D-26382 Wilhelmshaven, Germany; ramona.ohde@senckenberg.de (R.O.); ingrid.kroencke@senckenberg.de (I.K.); 4 Department of Biological Sciences, Institute of Ecology, Evolution and Diversity, Johann Wolfgang Goethe University, D-60439 Frankfurt am Main, Germany; 5 Faculty of Mathematics and Natural Sciences, Institute for Biosciences (IfBi), University of Rostock, D-18059 Rostock, Germany; stefan.forster@uni-rostock.de (S.F.); martin.powilleit@uni-rostock.de (M.P.)

**Keywords:** Natura 2000, Baltic Sea, North Sea, benthic habitats, invertebrates, fishing intensity, variability, environmental drivers, diversity, macrozoobenthos

## Abstract

**Simple Summary:**

This study illustrates a baseline biodiversity snapshot of macrofauna inhabiting the seafloor in German marine protected areas (MPAs) if the North and Baltic Seas in 2020–2022, before the full closure for bottom-contact fishing. While the closure is now in place in some MPAs, it is still planned for the near future in others. The analyses included different habitats in nine Natura 2000 MPAs. We provide essential data and comprehensive macrofauna species lists per area, relevant for the joint future conservation efforts and effective management. We explore environmental drivers of community structure and touch upon suggested effects of bottom-contact fishing in both geographic regions. Despite the expectation of more limited connectivity between MPAs in the Baltic Sea compared to the North Sea, the degree of community differentiation between MPAs was higher in the North Sea. Alpha diversity generally increased towards the open North Sea, and gamma diversity seemed comparable for these two regions. The Baltic Sea dataset unexpectedly contained a higher number of taxa, including Red List species. Achieving homogeneity of monitoring data and joint assessment even within one national program and biological compartment between different geographic regions, research institutions and fields remain challenging. This joint work appeals for flexible data sharing and prioritizing informal intersessional communication. Such a baseline is important for assessing future faunal changes.

**Abstract:**

The response of benthic habitats and organisms to bottom-contact fishing intensity is investigated in marine protected areas (MPAs) of the German EEZ in the North and Baltic Seas. We examined the current state of macrofauna biodiversity in 2020–2022. Comparative analysis for macrofauna (in- and epifauna) inhabiting nine Natura 2000 MPAs constitutes a baseline to assess the effects of bottom-contact fishing exclusion in the future. Aspects of spatial and temporal variability are briefly summarized and discussed. We provide a species list for each region, including 481 taxa, of which 79 were found in both regions, 183 only in the North Sea, and 219 only in the Baltic Sea. The Baltic Sea dataset surprisingly included higher numbers of taxa and revealed more Red List species. The share of major taxonomic groups (polychaetes, bivalves and amphipods) in species richness showed peculiar commonalities between the two regions. In the North Sea, multivariate analysis of community structure revealed significantly higher within-similarity and stronger separation between the considered MPAs compared to the Baltic MPAs. Salinity, temperature and sediment fractions of sand were responsible for over 60% of the variation in the North Sea macrofauna occurrence data. Salinity, mud fraction and bottom-contact fishing were the most important drivers in the Baltic Sea and, together with other considered environmental drivers, were responsible for 53% of the variation. This study identifies aspects of macrofauna occurrence that may be used to assess (causes of) future changes.

## 1. Introduction

Germany borders on two semi-enclosed seas, the North Sea [1] and the more continental brackish Baltic Sea [2]. These two seas, separated only by a few hundred kilometers of land mass (at the narrowest point only 33 km wide), possess distinct characteristics. Linked by the narrow passage of the Skagerrak and Kattegat [3], both seas offer a variety of ecosystem services necessary for humans and provide habitats or breeding grounds for hundreds of species including benthos [4,5]. Although they are both part of the Atlantic Ocean and are geographically close, they show remarkable differences in macrobenthic ecology [6].

The North Sea (NS), located between the coasts of Norway, Sweden, Denmark, Germany, the Netherlands, Belgium and the UK, is characterized by higher salinity and greater water movement. Powerful tides and strong currents ensure a high rate of water exchange with the open Atlantic. These dynamic conditions shape the diversity of habitats from the sandy coastal areas to the deep underwater trenches. In contrast, the Baltic Sea is semi-enclosed, surrounded by the coasts of Denmark, Sweden, Germany, Finland, Estonia, Latvia, Lithuania, Russia and Poland. The Baltic Sea (BS) is characterized by lower salinity and less water movement, barely affected by any tides [7]. This results in more stratified water and a limited exchange rate with the open ocean. These features have led to a unique evolution and adaptation of inhabiting organisms [8].

The large-scale distribution of the North Sea macrofauna communities was intensively studied since the last century (e.g., [9,10,11,12,13,14]). These studies confirmed a generally depth-based structure of three benthic zones: less than 50 m, between 50 and 100 m, and beyond 100 m [15]. Other environmental drivers of this zonation were sediment composition, depth, salinity, tidal patterns, sea surface temperature (SST), and primary productivity (PP). Comparable spatial studies of the ICES (International Council for the Exploration of the Seas) NS Benthos Survey (NSBS) in 1986 and the NS Benthos Project (NSBP) in 2000 (e.g., [10,12,16]) and recent studies of Fiorentino et al. [17] and Meyer et al. [14] identified four southeastern North Sea macrofauna communities at a depth < 50 m. These are the *Amphiura filiformis* community, the *Tellina (Fabulina) fabula* (or *Bathyporeia-Tellina*) community, the *Goniadella-Spisula* community, and the *Nucula nitidosa* community. Between 1986 and 2000, the spatial distribution of the four communities was stable [18]. However, structural changes within each of the southeastern North Sea macrofauna communities were since found in small-scale studies (e.g., [19,20,21]). These changes were often thought to be—directly or indirectly—driven by an increase in SST by 1.1 °C for the whole North Sea since 1950 [22] and about 2 °C for the southern North Sea [20].

Benthic macrofauna in the German waters of the Baltic Sea was systematically investigated since the 18th century (e.g., [23,24,25,26,27,28,29]). The distribution and dynamics of macrozoobenthos east of Fehmarn Belt were summarized in [30] based on data from 1839 to the 2000s. High temporal fluctuations in the occurrence, abundance, and biomass of macrozoobenthos were linked to (albeit natural irregular) saltwater intrusions and oxygen deficiency. The latter likely caused declines of some relict species, including the amphipods *Pontoporeia femorata* and *Monoporeia affinis*, the mussels *Macoma calcarea* and *Astarte* spp., or the isopod *Saduria entomon*. For other species, like the lugworm *Arenicola marina,* data suggested an eastward expansion. Relying on the findings by Zenkevitch [31], Schiewer [32] listed the most important species assemblages for the Baltic Sea, including the *Abra alba*-coenosis (with *Varicorbula gibba*, *Arctica islandica*, *Lagis koreni*, *Nephtys* spp., *Diastylis rathkei*, and *Ophiura albida*) dominating the western part, *Arctica*-*Astarte* assemblages found eastwards and *Macoma balthica*-coenosis dominating the shallow part of the Baltic Proper. Recent studies of spatial distribution on the large and medium scales suggest stability of community structure over time for some areas, higher fluctuations or even regime shifts due to species invasions for others, and increasing variability towards the entrance to the North Sea [33,34].

### 1.1. Habitat Protection

The German seas and MPAs therein are protected by various conservation measures to ensure their ecological integrity, biodiversity and the sustainable use of resources [35]. The German Exclusive Economic Zones (EEZ) of the North and Baltic Seas include ten nominated Natura 2000 sites within the EU Natura 2000 protected areas network [36]. The main international EU legislative drivers that regulate protection of endangered wild plants and animals in those special natural habitats are the Birds Directive and the Habitats Directive (Council Directive 92/43/EEC), as well as OSPAR and HELCOM regulations; nationally, they have the status of protected nature reserves [37]. The EU Marine Strategy Framework Directive (MSFD) divides benthic habitats into broad habitat types (BHTs) and, in accordance with the EU Commission, into biotope classes and other habitat types (OHT). In addition to the protected areas designated on the basis of the Habitats Directive, the OHTs thereby also include the particularly endangered biotope types such as species-rich gravel, coarse sand and gravel beds, or mudflats with drilling megafauna, based on OSPAR or national law (Section 30 BNatSchG).

In the North Sea, the “Dogger Bank” (DGB), “Borkum Reef Ground” (BRG), and the “Sylt Outer Reef–Eastern German Bight” (that comprise two sites included in this study: the “Sylt Outer Reef” (SAR) and the “Amrum Bank” (AMB)) cover an area of 7920 km^2^ (28% of the EEZ). In the Baltic Sea, the “Fehmarnbelt” (FB), “Kadetrinne” (KT) and “Pomeranian Bay—Rønne Bank” (including Adler Ground (AG), Odra Bank (OB) and Western Rønne Bank (RB)) have a total area of 2472 km^2^, which constitutes 55% of the EEZ [38,39].

### 1.2. MGF and BfN Monitoring Projects and Aims of This Study

Here, we aim to summarize baseline macrofauna biodiversity data gained within two research projects (MGF North Sea and MGF Baltic Sea, financed by the Federal Ministry for Education and Research (BMBF)) that investigate the effects of the exclusion of mobile bottom-contact fishing in MPAs of the German EEZ.

**Figure 1:**
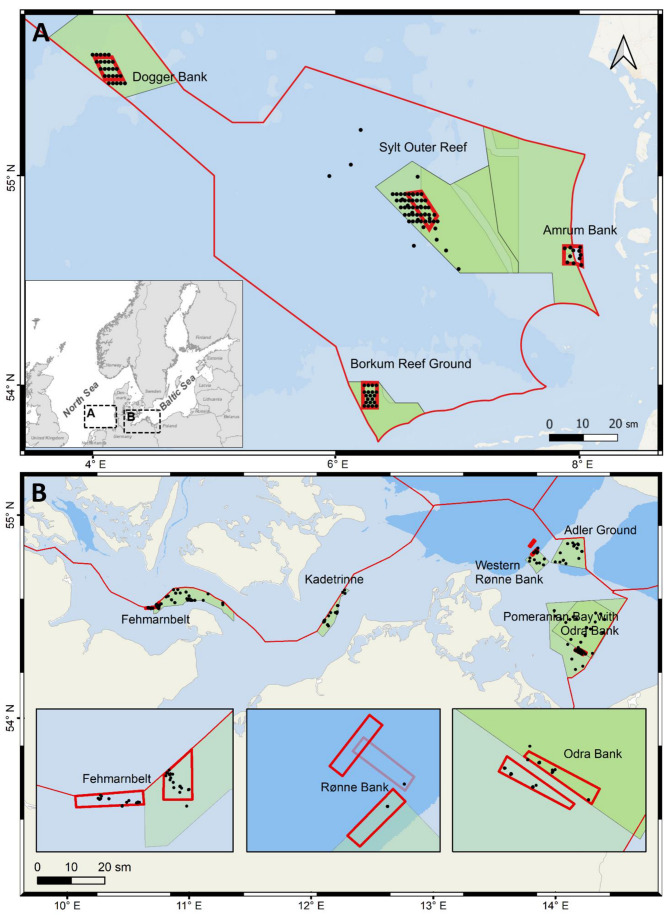
Maps of Natura 2000 sites (green polygons) and the MGF focus areas (thick red line boxes) in (**A**) the North Sea and (**B**) the Baltic Sea. The small grayscale inlet (inserted in (**A**)) shows a general view of the North and Baltic Seas. The thin red line marks the boundaries of the German Exclusive Economic Zone (EEZ). Black dots show the sampled stations. Focus areas in the Baltic Sea are zoomed in on the three small inlet maps. Dots inside the focus areas are stations sampled within the MGF Baltic Sea project, whereas other stations were mostly visited within the LEGRA and ATLAS projects. The half-transparent red line outlines the initial focus area in Rønne Bank, later shifted due to proximity to wind farms that inhibited later sampling. Intense green background outlines the future OB closure area if it will only take place in part of the MPA.

We complement it with external data to enhance spatial consistency across regions. In the North Sea, MGF-project data were collected from larger areas, while grid-based sampling was employed in designated MPAs (Figure 1A). In the Baltic Sea, sampling focused on specific areas within and outside future exclusion zones of MPAs (Figure 1B).

Macrofauna communities of the Western Baltic Sea and the North Sea have high exposure to natural and anthropogenic stressors [18,40] and especially to bottom-contact fishing [41,42,43,44,45,46]. Historically, in both regions, macrofauna monitoring and assessment are well-covered by established programs, such as projects in MPAs funded by the Federal Agency for Nature Conservation (BfN) like LABEL [47,48], CLUSTER and LEGRA [49], the project ATLAS (funded by the State Agency for Environment, Nature Conservation and Geology Mecklenburg-Vorpommern (LUNG MV) [50]), as well as long-term research studies (e.g., [18,51]).

Mobile bottom-contact fishing impacts on macrofauna have been studied in the North Sea in EU projects like IMPACT I-II [52,53], MAFCONS [54], and recently BENTHIS [55]; in ICES actions [37,56,57]; as well as in national projects (such as those named above or the recently launched CRANIMPACT, which investigates the effects of shrimp fishing on the seabed).

In the Baltic Sea, there have been no targeted studies since the 1990s [58,59,60]. The planned closure of MPAs for mobile bottom-contact fishing requires scientific evaluation of its efficiency. Such evaluation implies the development of optimal methods and monitoring concepts, particularly targeting those aspects that relate to potential changes, and is impossible without sufficient knowledge of the present standing stock and variability in macrofauna and understanding of its role in maintaining ecosystem services. Both MGF projects complement the existing monitoring programs.

Here, we do not aim for an explicit report of all the investigated macrofauna-related aspects but rather give a joint status quo summary to build upon and discuss the emerging peculiarities. We do aim to synthesize baseline macrofauna biodiversity data from MGF projects in German MPAs and evaluate impacts of mobile bottom-contact fishing and other environmental drivers on macrofauna.

## 2. Materials and Methods

### 2.1. The North Sea Case Study

#### 2.1.1. North Sea Study Areas

**Figure 2:**
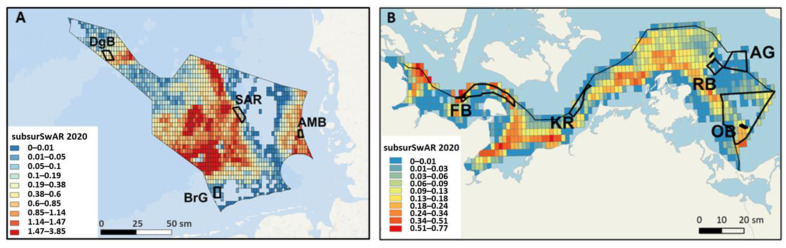
Overview of the mobile bottom-contact fishing intensity, (**A**) subsurface swept area ratio (>2 cm, subsurSwAR) in 2020 in the North Sea EEZ, based on ICES [62,63], and (**B**) subsurSwAR in 2020 in each 0.05° × 0.05°-degree c-square from ICES [64] data in the Baltic Sea EEZ; red = high mobile bottom-contact fishing intensity; blue = non or low mobile bottom-contact fishing intensity.

The sampled stations were located within or near the focus areas defined by the MGF North Sea project in order to be able to investigate the regions with strong anthropogenic influence before, during, and after the exclusion of mobile bottom-contact fisheries (Figure 1A and Figure 2A). We also included all sampled stations at the MPA Sylt Outer Reef (SAR). These MGF focus areas and the SAR stations allow a comparison of different subsamples with regard to the in- and epifauna, as well as temporal comparison with earlier collected data for certain areas [20,51,61].

The Dogger Bank (DGB) is a shallow, 300 km-wide sandbank in the central North Sea [20], interesting due to its faunal composition: in the north, it is characterized by species typical for the northern North Sea, while in the south, species typical for the southern North Sea are common [20,65]. However, the German MPA at DGB only covers a comparatively small part of the whole DGB. Borkum Reef Ground (BRG) is characterized by reef structures surrounded by sandbanks. It is located in the southern North Sea, relatively close to the coast of the East Frisian island of Borkum [66]. The Sylt Outer Reef (SAR) area shows a variety of sediment structures with reefs, gravel areas and sandbanks [67]. The Amrum Bank (AMB) is mainly characterized by sandy substrate. In addition, due to strong wind conditions, the sandbank is used for wind turbine installation. Exclusion of mobile bottom-contact fishing in 2023 took place in a large part of SAR and the entire area of BRG.

#### 2.1.2. North Sea Data Collection

A total of 150 stations were sampled within the four study areas in the North Sea (Figure 1A) with RV “Senckenberg” in 2020–2022 in order to study the in- and epifauna biodiversity along the gradients of decreasing bottom-contact fishing intensity (Table 1).

The sampling for in- and epifauna took place at the same research cruise only in 2020 at the MPA BRG (14 stations). During this research cruise at these stations the infauna sampling was performed, and next, the epifauna sampling was performed. The other stations were sampled in separate cruises. At the MPA DGB 20 stations sampled in 2021 had the same location but were visited on different cruises in different months (Table 1). Only four stations in 2020 at the MPA SAR had the same locations for the in- and epifauna sampling (Table 1). For the infauna, two replicate samples were collected with a 0.1 m^2^ van Veen grab at each station and were sieved through a 1 mm mesh size. The samples were preserved on board in a 4% buffered formaldehyde-seawater solution. Retained material was identified to the lowest possible taxonomic level. The taxonomy (also for the Baltic Sea) was harmonized following the World Register of Marine Species [68].

During epifauna sampling, at each station first, the water temperature and salinity were determined using a CTD probe (Sea and Sun technologies). After measurement of water parameters, a ring dredge (diameter 1 m, mesh size 1 cm^2^) was lowered to the seafloor for sampling the main taxa of the in- and epifauna. The dredge was slowly pulled by the ship in a constant direction for 3–5 min (depending on the prevailing sediment). The ring dredge penetrated about 5 cm into the sediment of the seafloor (also depending on the sediment type) so that after retrieval, the main in- and epifauna could first be documented photographically, sorted and identified to the finest taxonomic level possible. The identified species were recorded in a presence/absence matrix and then released directly back into their natural habitat to ensure their survival. Additionally, the epifauna was subsequently sampled using a 2 m beam trawl (rump mesh size 1 cm^2^). The beam trawl was lowered to the seafloor and then towed in one direction at 2 knots over a distance of 1 nautical mile (=1.85 km). The sample was then documented photographically on board, and the fine fraction (>1 mm) was separated from the larger sieve fraction (<1 cm) using a sieve barrel. After sorting and identification, caught species were quantitatively recorded in the case of non-colonial forms. The sieve fractions (>1 mm) of the beam trawl sampling and species that could not be determined directly on board were fixed in 96% ethanol or in a ~5% formaldehyde-seawater solution to ensure later final determination in the Senckenberg Research Institute’s laboratory.

### 2.2. The Baltic Sea Case Study

#### 2.2.1. The Baltic Sea Study Areas

The three MGF Baltic Sea focus areas (Figure 1B) selected in the Fehmarnbelt (FB), Rønne Bank (RB) and Odra Bank (OB) are characterized by different sediments. While the FB focus area is located on muddy sediment with a fine sand component, the area selected at RB is covered by fine, organically rich mud, and the OB focus area is a typical sand bank. Moreover, they are also home to different communities due to the gradient of salinity, which, in the Baltic Sea, decreases sharply from west to east. Thus, different responses to bottom-contact fishing intensity and termination thereof are expected in the three areas. Not all MPAs in the Baltic Sea are equally affected by bottom-contact fishing. Two MPAs, Kadetrinne (where highly intensive ship traffic takes place) and the Adlergrund, both characterized by reef structures (not favored by trawling fishers due to the risk of fishing gear damage), were excluded from the MGF investigation as less relevant in order to keep the efforts feasible (see Figure 2B). However, on the larger scale, the condition of these reef MPAs and inhabiting benthic fauna was annually monitored within the LEGRA project.

#### 2.2.2. Data Collection: Baltic Sea

A total of 222 stations were sampled in the Baltic Sea MPAs in 2020–2022, and 35 more stations in close vicinity (Table 2, Figure 1B). At each station and visit, for quantitative macrofauna data, three replicate samples were commonly collected with a 0.1 m^2^ van Veen grab (weight about 75 kg, sediment penetration depth of up to 15–20 cm) and washed through a 1 mm sieve. Remaining animals were preserved in a 4% formaldehyde-seawater solution buffered with marble chippings; material was sorted in the laboratory at the Leibniz Institute for Baltic Sea Research, Warnemünde, with a stereomicroscope and identified to the lowest possible taxonomic level. Organisms were counted and weighed to obtain estimates of species abundance and biomass per square meter. At three specific areas—FB, RB, and OB—we identified the “key species” defined here as those having a substantial contribution to biomass, an extended lifespan, a high potential for bioturbation, and a pivotal role in the local food web [69,70].

We also took 0.00785 m^2^ sediment core samples with a multicorer. The number of cores per station varied from one to six. Cores were sliced for macrofauna vertical distribution (using 7 intervals of 0–2, 2–4, 4–6, 6–8, 8–10, 10–15, and >15 cm sediment depth) and each slice was sieved separately with a 0.5 mm sieve.

Additionally, the Kieler Kinderwagen dredge was used to qualitatively assess quick-moving, rare or large species [71]. The dredge has a 92 cm inner opening, and 5 mm mesh; it was towed with 1 knot over the ground for about 1 min (=31 m) in mud and 5 min in sand (=155 m), penetrating the sediment to roughly 5 cm in mud and only scraping the sediment surface in sand.

Epifauna in the studied habitats was additionally investigated using an underwater video system (only a hand-held SeaViewer HD camera could be used on MGF transects due to logistical limitations, whereas in LEGRA campaigns, the BaSIS system that is suitable for gathering quantitative coverage data [72] was also applied).

### 2.3. Environmental Drivers, Mobile Bottom-Contact Fishing Data and Statistical Analysis

#### 2.3.1. Temperature, Salinity, and Sediment Data

CTD near-bottom water measurements were conducted at each location before biological sampling in order to obtain relevant abiotic parameters (including near-bottom water temperature and salinity (for North Sea and Baltic Sea) and oxygen concentrations (only for Baltic Sea)).

A surface sediment sample (upper 2 cm) was taken from one additional grab replicate at each location for later sediment granulometry. The North Sea sediment samples were sieved using a 63 µm mesh size to determine the mud content (<63 µm, in %). The shell content (>2000 µm, in %) was determined by wet dry sieving over a 2 mm mesh. In addition, the % of gravel debris was measured. For the Baltic Sea samples, dry sieving was used for sands, and a Mastersizer 3000 was used for finer sediments.

For the Baltic Sea, mean near-bottom temperature values for 2010–2020 available from the GETM model [73] were extracted using ArcGIS for each sampling location to illustrate general longer-term conditions.

#### 2.3.2. Bottom-Contact Fishing Intensity

Data describing mobile bottom-contact fishing intensity originated from ICES for both the North Sea [62,63] and the Baltic Sea regions [64,74]. The intensity of bottom-contact fishing was calculated based on VMS and linked logbook data submitted by EU member states to ICES and aggregated consistently across years and quarters for 2016–2020 for the North Sea and 2016–2021 for the Baltic Sea period. Intensity is expressed in either kilowatt fishing hours (kwfhr) or as surface or subsurface swept area ratio (surSwAR or subsurSwAR) at the spatial resolution of c-square with the extension of 0.05° × 0.05° degrees. A SwAR value of 1 implies that the sediment of the entire area was trawled once or, e.g., that half of the area was swept over twice within a period of time (here recalculated to multiannual values). Surface SwAR (surSwAR) reflects the potential impacts on benthic epifauna, considering the surface penetration depth (<2 cm) of the gear components. The impact on benthic infauna is reflected in the Subsurface SwAR (subsurSwAR), considering the subsurface penetration depths (≥2 cm) of each gear, assuming no differences across sediment types [75].

#### 2.3.3. Statistical Analysis

To avoid the bias related to the differences in sampling methods and efforts applied in the studied regions, we focused here on multivariate statistical techniques, such as ordination, to explore patterns in species composition and occurrence within each area rather than abundance.

A non-metric multidimensional scaling (nMDS) and similarity profile analysis (SIMPROF) were accomplished for each MPA, based on a Bray–Curtis resemblance matrix of the Presence/Absence transformed abundance data, separately for each region, using PRIMER 6 for the North Sea and PRIMER 7 for the Baltic Sea Data. The SIMPROF analysis is a permutation test analyzing the statistical significance of groups. Characteristic taxa for each MPA were identified by similarity percentage analysis (SIMPER), using the Bray–Curtis similarity matrix. The defined clusters (representing the MPAs) were confirmed by Analysis of Similarities (ANOSIM), which is a permutation test, analyzing the statistical significance of a priori divided clusters. ANOSIM reveals a global R for the whole dataset and a pairwise R, testing between the clusters [76].

For the North Sea statistical analyses, we only used the data from the stations for which we had abundance data for both in- and epifauna. For the MPA SAR there were only four stations sampled in 2020 for which both data sets were available. In the MPA BRG, 14 stations were sampled in 2020, and in the MPA DGB, 20 stations in 2021. In the Baltic Sea, all stations were included, since sampling was targeting all macrofauna without distinguishing between in- and epifauna.

To determine the set of environmental drivers that best explain the variation of benthic macrofauna at each MPA, we performed a distance-based linear model permutation test (DistLM) based on a significant RELATE analysis employing the routine from the software PRIMER 6 with PERMANOVA+ add-on [77]. Predictor variables were subjected to a sequential stepwise selection procedure using Akaike’s information criterion with a correction for finite sample size (AICc). To calculate resemblance in DistLM, the Bray–Curtis similarity was used. We included several environmental drivers (Table 3) that can affect macrofauna and the available corresponding mobile bottom-contact fishing intensity data at each location as additional independent variables. Predictor pairs were tested for collinearity.

For both the NS and BS, based on a correlation threshold of 0.8 (with higher values suggesting multicollinearity) and the marginal test results, only subsurface SwAR was retained in DistLM as the most influential out of three initially considered fishing intensity parameters. For the BS, some variables (% mud, % gravel and subsurface SwAR) were square root transformed to remove right-skewness in the raw data in case it was observed on Draftsman’s plots. For the NS, only the subsurface SwAR was square root transformed.

## 3. Results

### 3.1. Biodiversity and Community Analysis

#### 3.1.1. Species Richness and Major Groups

A total of 481 taxa were found in all nine MPAs of the Baltic and North Sea during 2020–2022 (see full taxa list in Supplementary Material S1; there, all the scientific names are provided with authorities, whereas for the sake of readability, authorities are mostly omitted here in the main text). The 481 taxa belonged to the phylum Annelida (162 taxa), Arthropoda (126 taxa), Mollusca (100 taxa), Cnidaria (33 taxa), Echinodermata (19 taxa), Bryozoa (16 taxa), Porifera (7 taxa), Chordata (6 taxa), Nemertea (6 taxa), Priapulida (3 taxa), Phoronida (1 taxon), Platyhelminthes (1 taxon) and Entoprocta (1 taxon).

Only in the North Sea MPAs, 183 of the 481 (38.0%) taxa were found, for example the polychaete *Aonides paucibranchiata*, the crustacean *Urothoe elegans*, or the bivalve *Gari fervensis*.

Exclusively in the Baltic Sea MPAs, 219 of the 481 (45.5%) taxa were found. The polychaete *Dipolydora quadrilobata*, the gastropod *Alvania punctura*, and the echinoderm *Ekmania barthii* were examples of such taxa found in the MPAs of the Baltic Sea but not in the North Sea MPAs.

79 of the 481 (16.4%) taxa occurred in the North Sea and in the Baltic, for example the polychaete *Eteone longa*, the crustacean *Pagurus bernhardus*, or the gastropod *Aporrhais pespelecani*. Only one of the 481 (0.2%) taxa was found in all of the nine MPAs: the crustacean *Crangon crangon*.

180 of the total of 481 (37.4%) taxa were found in only one of the nine MPAs in the North and Baltic Sea. For example, the crustacean *Tryphosites longipes* was present only in the SAR, and the echinoderm *Echinocardium flavescens* only in the AMB in the North Sea, whereas the mollusc *Lamprops fasciatus* was found only in the FB, and the gastropod *Ecrobia ventrosa* only in the OB in the Baltic Sea.

##### North Sea

**Figure 3:**
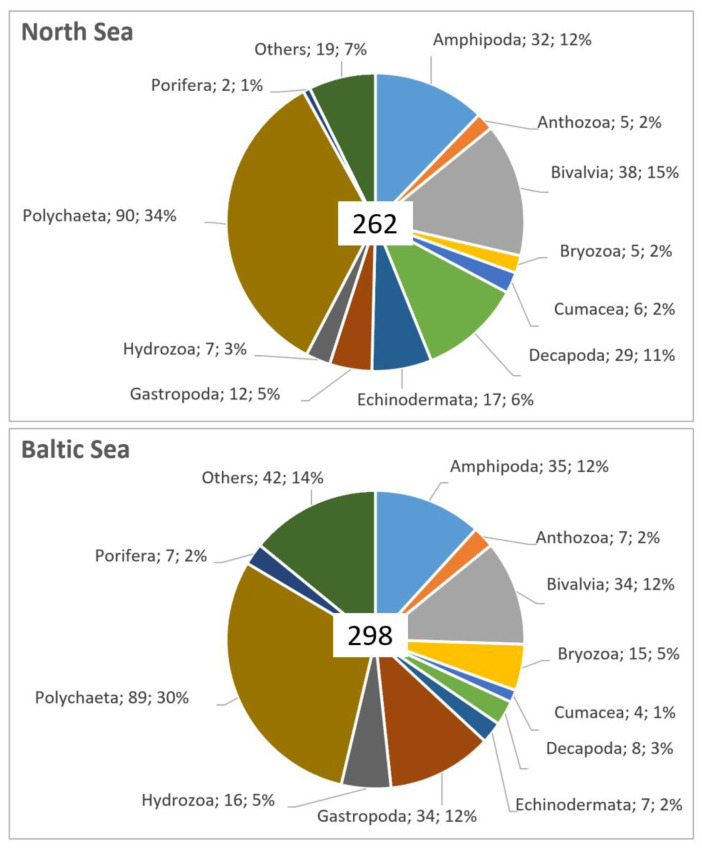
Number and (after semicolon) percentage of taxa found per group in the MPAs in the North Sea (**upper pane**) and the Baltic Sea (**lower pane**). The groups used here in order to facilitate the summary should be rather considered as functional, i.e., not strictly taxonomic, as they vary in rank ranging from Phylum to Order level. In the North Sea MPAs (upper pane), the category “other” includes Isopoda (4), Cirripedia (3 taxa), Nemertea (2), Sipuncula (2) and single taxa of Ascidiacea, Leptocardii, Oligochaeta, Phoronida, Platyhelminthes, Priapulida, Pycnogonida and Tanaidacea. In the Baltic Sea MPAs (lower pane), the category “other” includes Oligochaeta (6), Isopoda (5), Mysida (5), Nemertea (5), Ascidiacea (4 taxa), Cirripedia (4), Priapulida (2), Pycnogonida (2), Tanaidacea (2) and single taxa of Arachnida, Entoprocta, Hirudinea, Leptocardii, Phoronida, Platyhelminthes and Polyplacophora.

A total of 262 taxa were found in the four MPAs in the NS during 2020–2022 (Supplementary Material S1). The 262 taxa belonged to the phylum Annelida (93 taxa), Arthropoda (76 taxa), Mollusca (50 taxa), Echinodermata (17 taxa), Cnidaria (12 taxa), Bryozoa (5 taxa), Chordata (2 taxa), Nemertea and Porifera (2 taxa each), as well as 1 taxon each of Phoronida, Platyhelminthes and Priapulida (Figure 3).

The most taxa were found in the SAR with 187 taxa (Table 3). Only 17 of the 262 (6.5%) taxa were found in all four MPAs of the North Sea, for example, the polychaete *Nephtys hombergii* and the decapod *Corystes cassivelaunus*. 106 of the 262 taxa (40.5%) were present in only one of the four MPAs of the North Sea. The holothurian *Leptosynapta inhaerens*, the gastropod *Epitonium clathrus* and the decapod *Goneplax rhomboides* appeared only in the MPA SAR. In the MPA BRG, the polychaete *Hesionura elongata* and the bivalve *Lutraria lutraria* occurred exclusively. The echinoderm *Amphipholis squamata*, the gastropod *Euspira montagui* and the polychaete *Hydroides norvegica* were only found in the MPA DGB (see full species list in Supplementary Material S1).

##### Baltic Sea

A total of 298 taxa were found in the five MPAs of the Baltic Sea during 2020–2022 (Supplementary Material S1). These taxa belonged to the phylum Annelida (96 taxa), Mollusca (69 taxa), Arthropoda (66 taxa), Cnidaria (23 taxa), Bryozoa (15 taxa), Echinodermata (7 taxa), Porifera (7 taxa), Chordata (5 taxa), Nemertea (5 taxa) and one taxon each of Phoronida, Platyhelminthes and Entoprocta (Figure 3).

The most taxa were found in the focus area FB with 264 taxa (Table 3). Only 19 (6.4%) of taxa were found in all five MPAs of the Baltic Sea: for example, the polychaete *Hediste diversicolor* and the bryozoan *Einhornia crustulenta*. 145 of the 298 taxa (48.7%) were present in only one of the five MPAs. The bivalve *Tellimya ferruginosa* and the amphipod *Aora gracilis* appeared in the MPA FB only. The gastropod *Theodoxus fluviatilis*, the tanaid *Heterotanais oerstedii* and the fish leech *Piscicola* sp. occurred exclusively in the MPA AG. The polychaete *Marenzelleria neglecta* and the crustacean *Rhithropanopeus harrisii* were only found on the OB (see full species list in Supplementary Material S1).

#### 3.1.2. Community Structure

##### North Sea

**Figure 4:**
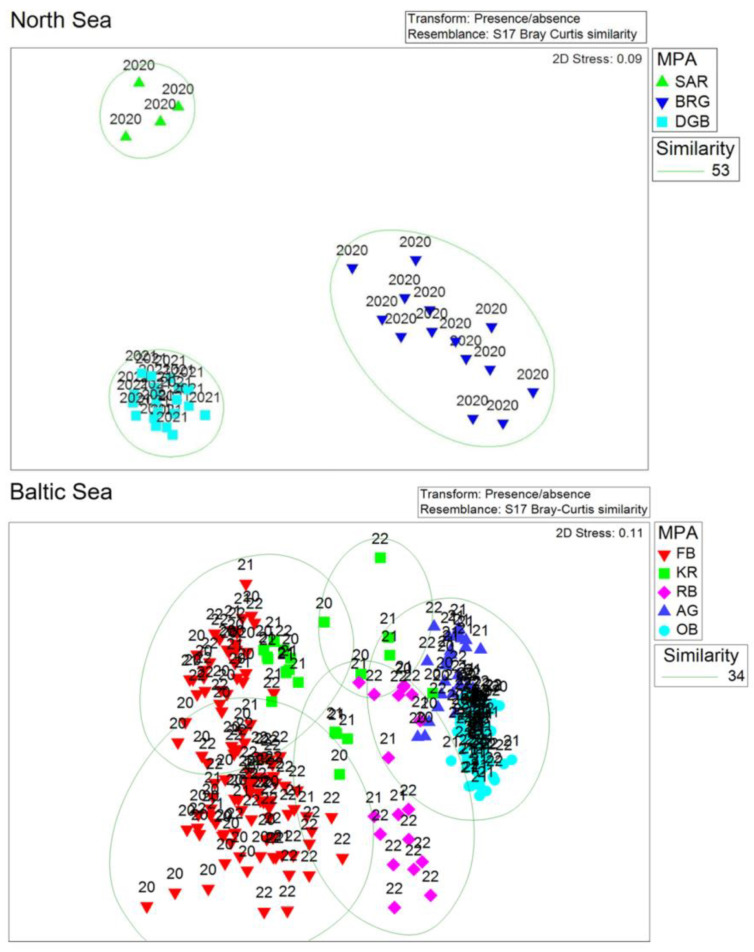
Multidimensional scaling (MDS) plots for the North Sea and the Baltic Sea areas based on presence/absence transformed data. The North Sea plot only includes stations where both data sets for in- and epifauna were available. Labeling is according to the MPAs and the sampling years.

**Figure 5:**
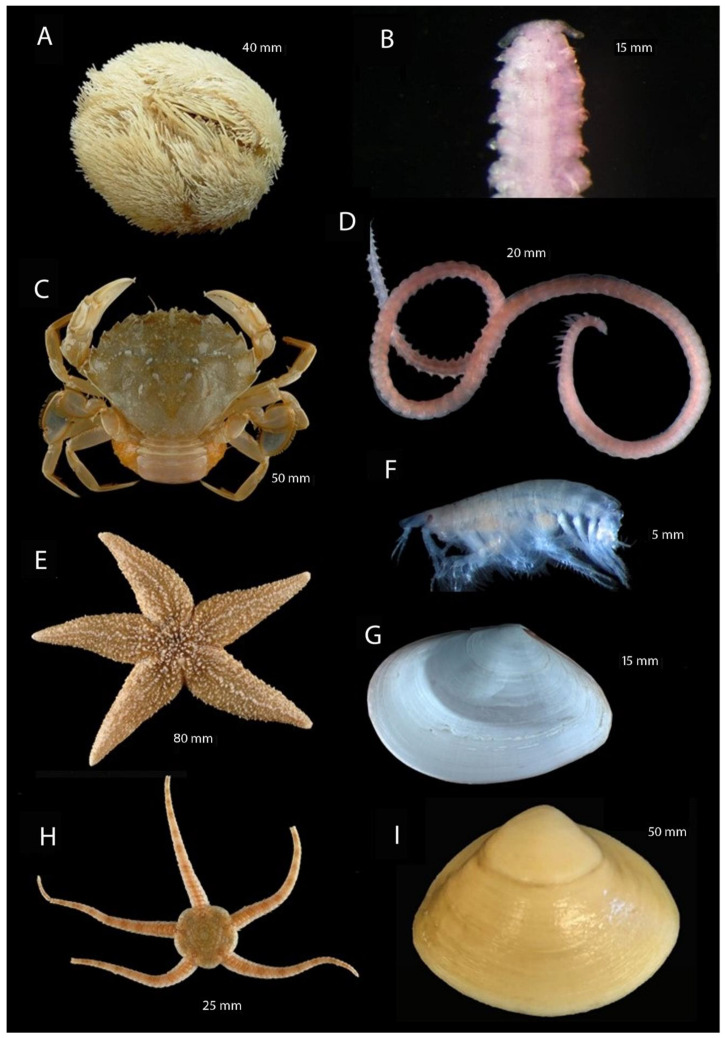
Most common species in the North Sea MPAs. (**A**) *Echinocardium cordatum* (Pennant, 1777), (**B**) *Spiophanes bombyx* (Claparède, 1870), (**C**) *Liocarcinus holsatus* (Fabricius, 1798), (**D**) *Aonides paucibranchiata* Southern, 1915, (**E**) *Asterias rubens* Linnaeus, 1758, (**F**) *Bathyporeia elegans* Watkin, 1940, (**G**) *Abra alba* (W. Wood, 1802), (**H**) *Ophiura ophiura* (Linnaeus, 1758), (**I**) *Spisula solida* (Linnaeus, 1758). Indicated sizes are approximate total lengths (of longest dimension) for all species, with two exceptions: for *L. holsatus* (**C**), the value corresponds to carapace length, and for *O. ophiura*, the disc diameter is specified. These sizes were measured with calipers and are provided only for visualization and to show scale differences between species; they are not relevant for any other reported results.

The analysis of community structure based on presence/absence transformed data for the NS showed distinct differences between the MPAs (Figure 4, upper pane). The MPAs in the North Sea can be significantly separated in terms of the macrofauna (ANOSIM: R-value = 0.991; *p*-value = 0.001), at least based on stations where in- and epifauna data were available. Some exemplary most frequent species found in the study region are shown in Figure 5.

The SIMPER analysis revealed the mean similarity of MPAs (Table 4). The MPA SAR had a mean similarity of 62.3%. It was mainly characterized by the bivalve *Abra alba,* the echinoderm *Amphiura filiformis*, the echinoderm *Astropecten irregularis,* the bivalve *Chamelea striatula*, and the decapod *Corystes cassivelaunus*. The mean similarity in the MPA BRG was 57.5%. Characterizing taxa were the polychaete *Aonides paucibranchiata*, the echinoderm *Astropecten irregularis*, *Ensis* spp. bivalves, the polychaete *Lanice conchilega*, and the swimming crab *Liocarcinus holsatus*. The MPA DGB had a mean similarity of 67.5% and was mainly characterized by the echinoderm *Amphiura filiformis*, Cnidaria (Anthozoa indet.), the amphipod *Aora gracilis*, as well as by the echinoderms *Asterias rubens* and *Astropecten irregularis*.

##### Baltic Sea

**Figure 6:**
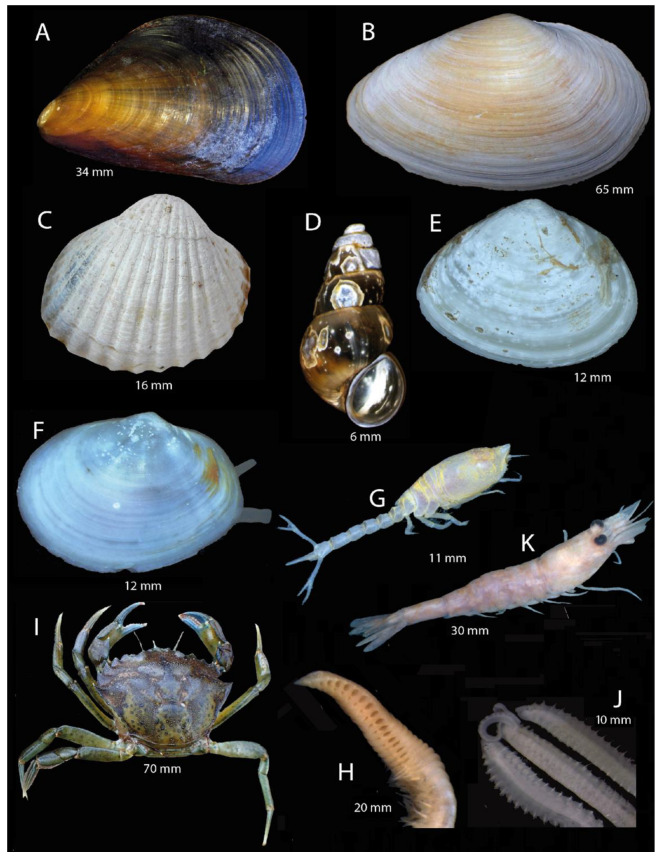
Most common species in the Baltic Sea MPAs. Size measures for each species are given in mm. (**A**) *Mytilus edulis* Linnaeus, 1758, (**B**) *Mya arenaria* Linnaeus, 1758, (**C**) *Cerastoderma glaucum* (Bruguière, 1789), (**D**) *Peringia ulvae* (Pennant, 1777), (**E**) *Macoma balthica* (Linnaeus, 1758), (**F**) *Abra alba* (W. Wood, 1802), (**G**) *Diastylis rathkei* (Krøyer, 1841), (**H**) *Scoloplos armiger* (Müller, 1776), (**I**) *Carcinus maenas* (Linnaeus, 1758), (**J**) *Pygospio elegans* Claparède, 1863, (**K**) *Crangon crangon* (Linnaeus, 1758). Indicated sizes are approximate total lengths (of longest dimension) for all species but I (for *C. maenas*, the value corresponds to carapace length). These sizes were measured with calipers and are provided only for visualization and to show scale differences between species; they are not relevant for any other reported results.

The analysis of community structure for the BS based on presence/absence data (Figure 4, lower pane) showed less difference between MPAs compared to the NS dataset. The MPAs in the BS could still be significantly separated in terms of the macrofauna (ANOSIM: R-value = 0.784; *p*-value = 0.001). Some exemplary most frequent species found in the study region are shown in Figure 6.

The SIMPER analysis revealed the mean similarity of each MPA (Table 5). The FB MPA had the lowest mean similarity of 38.8% among the Baltic Sea MPAs. In terms of presence/absence, FB was mainly characterized by the polychaetes *Aricidea suecica* and *Scoloplos armiger*, the echinoderm *Ophiura albida,* the bivalve *Varicorbula gibba* and the cumacean *Diastylis rathkei*. The mean similarity in the KR MPA was 46.9%. Characterizing taxa were the bivalve *Mytilus edulis,* the gastropod *Peringia ulvae,* the polychaetes *Bylgides sarsi* and *Pygospio elegans,* and the bryozoan *Eucratea loricata.* The MPA RB had a mean similarity of 49.2% and was mainly characterized by the bivalve *Macoma balthica*, the gastropod *P. ulvae,* the polychaete *S. armiger,* the cumacean *Diastylis rathkei* and the amphipod *Pontoporeia femorata*. Within the stations of AG MPA, a mean similarity of 60.2% was observed, mainly driven by the bivalve *M. edulis,* the gastropod *P. ulvae,* the amphipod *Gammarus salinus,* the bryozoan *Einhornia crustulenta,* and the polychaete *P. elegans*. In the OB MPA, the mean similarity was 64%, and characteristic species were the gastropod *P. ulvae*, the polychaete *P. elegans*, the oligochaetes of subfamily Tubificinae, as well as the bivalves *M. edulis* and *Mya arenaria.*

Additionally, within the three MGF focus areas in the Baltic Sea, it is worth noting the “key species”, which we defined as biomass-dominant bivalves possessing a long lifespan, playing a crucial role in the local food web, and making significant contributions to bioturbation. For FB, it is the ocean quahog *Arctica islandica*, with biomasses > 90% of the total macrozoobenthos biomass; for RB, it is the Baltic tellin *Macoma balthica*, with biomasses of about 23% of the total biomass. For OB, there are two key species: the sand gaper *Mya arenaria* as well as *M. balthica*, with biomasses of about 54% and 18% of the total biomasses, respectively.

### 3.2. Variation Explained by Environmental Drivers and Trawling Intensity

#### 3.2.1. North Sea

**Figure 7:**
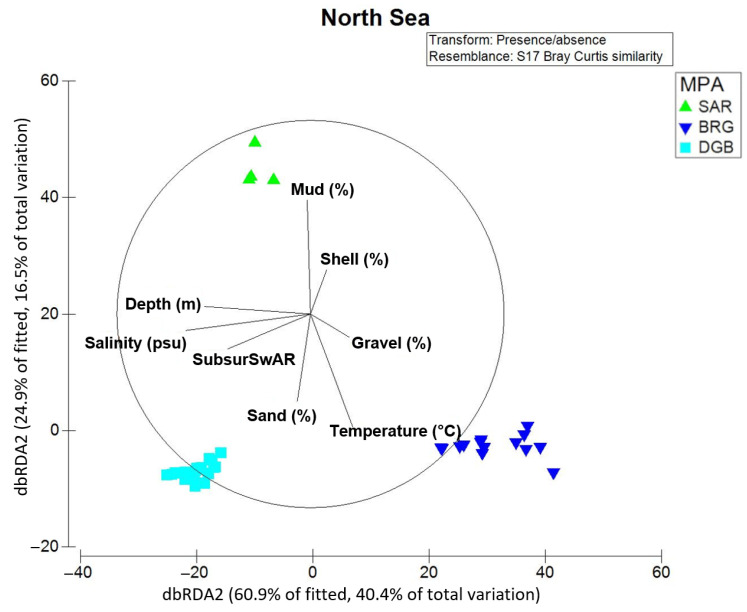
dbRDA ordination of stations in the North Sea MPAs along environmental (depth (m), sediment parameters (shell fraction > 2 mm, sand fraction < 2 mm to >0.063 mm, mud fraction < 0.063 mm, and gravel fraction), temperature (°C) and salinity (psu)) and anthropogenic (bottom-contact fishing expressed as subsusSwAR) drivers. Labeling according to the MPAs.

The results of the dbRDA (Figure 7) show the relationship between the environmental drivers that best explain the variability in the macrofauna communities in the three North Sea MPAs. The RELATE analysis revealed a significant relation of the environmental drivers and bottom-contact fishing to the presence/absence macrofauna data with a Rho of 0.815 (significance level of 0.1%).

In the North Sea, the set of considered abiotic predictors explained together 66.3% of the total variation in the presence/absence macrofauna data. Based on results of the marginal test, salinity explained 39.7%, the depth (m) of the stations was responsible for 38.7% of changes in community structure, and the bottom-contact fishing explained 33.6% (SubsurSwAR) of variation in presence/absence data (see “DistLM results North Sea” tab in the Supplementary Material S1). In the sequential test (see “DistLM results North Sea” tab in the Supplementary Material S1), salinity (psu) as the most important predictor was followed by temperature and fraction of sand that explained an additional 20.5% of variation in macrofauna.

#### 3.2.2. Baltic Sea

**Figure 8:**
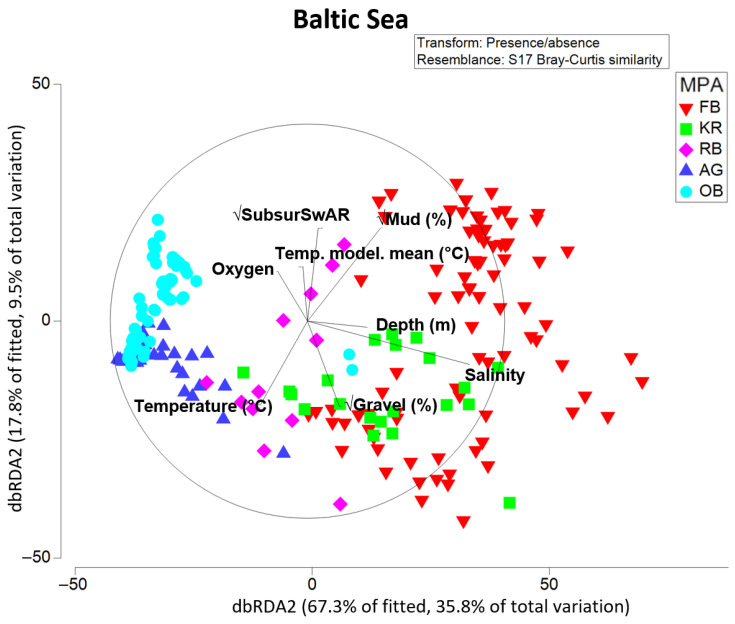
dbRDA ordination of stations in the Baltic Sea MPAs along environmental and anthropogenic (bottom-contact fishing expressed as subsurSwAR) drivers. Labeling according to the MPAs.

The results of the dbRDA (Figure 8) show the relationship between the environmental drivers that best explain the variation in the macrofauna composition in the five sampled Baltic Sea MPAs. Here, the set of considered abiotic predictors explained 53.1% of the total variation in the presence/absence macrofauna data. The dbRDA1 was mainly driven by salinity and % mud in sediment, and the dbRDA2 by bottom-contact fishing (subsurface SwAR), % mud, % gravel, and measured near-bottom temperature. Based on results of the marginal test, salinity of the near-bottom water alone was responsible for 33.3% of changes in community structure, mud content in sediments explained 24.3%, and among fishing parameters (that were highly correlated with each other), subsurface SAR explained 13.7% of variation in presence/absence data. In the sequential test (see “DistLM results Baltic Sea” tab in the Supplementary Material S1), bottom-contact fishing expressed in subsurface SwAR was the second most important predictor, explaining an additional 8.2% of variation in macrofauna. Modeled near-bottom water temperature and % gravel showed the least direct effect on community structure among considered predictors (based on the marginal test), but were still significant and retained in the final model.

### 3.3. Endangered Species

In total, 110 of the 481 (22.9%) taxa found in all considered MPAs of the North and the Baltic Seas were at or near risk of various degrees of extinction (Table 6). In addition, 47 of the 481 (9.8%) taxa are endangered with the status “Threat of unknown Extent” according to the Red List based on [78,79]. Some examples were the chordate *Branchiostoma lanceolatum*, the echinoderm *Astropecten irregularis*, the polychaete *Fabriciola baltica* and the bivalve *Musculus discors*.

#### 3.3.1. North Sea

Fifty-nine of the 262 taxa found in the North Sea were at or near risk of various degrees of extinction, accounting for 22.5% of the taxa (Table 6). Five taxa are endangered with the status “Highly Threatened”: the gastropod *Buccinum undatum*, the polychaete *Sabellaria spinulosa*, and the bivalves *Ensis ensis*, *Mya truncate*, and *Spisula elliptica*. The crustaceans *Ebalia tumefacta* and *Lepas anatifera*, the bivalves *Arctica islandica*, *Ensis magnus*, and *Goodallia triangularis*, and the cnidarian *Alcyonium digitatum* are endangered with the status “Threatened”. The status “Near Threatened” is allocated to the polychaete *Polygordius appendiculatus*, the decapod *Galathea intermedia,* the amphipod *Megaluropus agilis*, the bryozoan *Membranipora membranacea*, the echinoderms *Amphipholis squamata* and *Ophiothrix fragilis* and the gastropod *Acteon tornatilis*.

#### 3.3.2. Baltic Sea

In total, 67 of the 298 taxa found in the Baltic Sea were at or near risk of various degrees of extinction, accounting for 22.5% of the taxa (Table 6). Two taxa endangered with the status “Threatened with Extinction” are the cnidarian *Halcampa duodecimcirrata* and the bivalve *Macoma calcarea*. The bivalves *Modiolus modiolus* and *Mya truncata*, the gastropods *Boreotrophon truncatus* and *Buccinum undatum* and the polychaete *Euchone papillosa* are endangered with the status “Highly Threatened”. The status “Threatened” is allocated to the amphipod *Monoporeia affinis*, the bivalves *Arctica islandica* (though not uncommon in the typical *Arctica* community in aphotic muddy sediment) and *Astarte montagui*, the hydrozoan *Halitholus yoldiaearcticae* and the polyplacophoran *Lepidochitona cinerea*.

## 4. Discussion

### 4.1. Species Richness and Composition in MPAs

We found in a total of 481 taxa in all nine MPAs in the North and the Baltic Seas (see full taxa list in Supplementary Material S1). Contrary to our expectation and the literature [40], 37.9% of the taxa were found exclusively in the North Sea MPAs, while 45.8% of our taxa were found exclusively in the Baltic Sea MPAs, and only 16.3% were shared by the two seas. According to the annotated checklist from Zettler et al. [40], 36.6% of taxa were shared between two seas, 48.7% occurred only in the NS, while 14.7% occurred only in the BS. Among taxa found only in the NS, both in Zettler et al. [40] and in our study are the polychaete *Aphrodita aculeata* and *Nephtys cirrosa*, the echinoderm *Echinocardium cordatum*, the decapod *Necora puber*, the amphipod *Megaluropus agilis* and the bivalve *Dosinia lupinus*. Example taxa specific to the BS in both studies are the bivalve *Astarte borealis*, the polychaetes *Fabriciola baltica* and *Spio arndti*, the gastropod *Lacuna parva* and the amphipod *Pontoporeia femorata*.

Some examples of species found in both areas and confirmed by both studies are the echinoderms *Amphiura filiformis* and *Echinocyamus pusillus*, the crustaceans *Carcinus maenas* and *Pagurus bernhardus*, the polychaetes *Eteone longa* and *Lanice conchilega* and the bivalves *Arctica islandica* and *Kurtiella bidentata*.

Multivariate analysis of the North Sea macrofauna data in our study revealed low similarity between the three considered MPAs, DGB, SAR and BRG. Based on ring dredge and beam trawl data from 33 stations in SAR and BRG sampled in 2020, Hahn et al. [80] published a checklist of benthic fauna that comprised 99 species from the phyla Mollusca, Arthropoda, Echinodermata, Annelida, Cnidaria, and Bryozoa (listed according to descending species number per group). In line with our results, Hahn et al. [80] also reported clear separation of species composition between the two areas. In contrast to our results, the higher species diversity found at BRG (compared to SAR) in that dataset was associated to lower bottom-contact fishing pressure. In our study, community composition within one area was more similar in DGB and SAR compared to the BRG. In the southeastern North Sea, differences in macrofauna biodiversity, intensively studied since the last century (e.g., [9,10,11,12,13,14,81]), are well represented by the four distinct macrofauna communities already mentioned in the introduction. The macrofauna communities of the DGB, BRG and SAR are assigned to the *Bathyporeia-Tellina,* the *Goniadella-Spisula*, and the *Amphiura-filiformis* communities, respectively [17]. According to Fiorentino et al. [17] taxa identified as characteristic for the *Bathyporeia-Tellina* community are *Bathyporeia elegans*, *Lanice conchilega*, *Tellina (Fabulina) fabula* and *Spiophanes bombyx*. These macrofauna species were also found in DGB MPA in our study. Among characteristic taxa of the *Goniadella-Spisula* community, Fiorentino et al. [17] listed *Aonides paucibrachiata*, *Branchiostoma lanceolatum*, *Pisione remota* and *Echinocyamus pusillus*, reported in our list for BRG MPA. In agreement with our results for SAR MPA, taxa listed as characteristic for the *Amphiura filiformis* community, apart from the name-giving species, were *Kurtiella bidentata*, *Nucula nitidosa* and *Phaxas pellucidus*.

Multivariate analysis for the Baltic Sea macrofauna suggested less distinct and therefore more similar community structure between the five MPAs compared to those in the North Sea. This is likely due to the inclusion of multiple habitats and biotopes with patchy distribution in each of the German Baltic Sea MPAs [50], and due to less distinct boundaries and gradual ecological transitions along environmental gradients between the adjacent communities. In agreement with previously reported increasing variability towards the entrance to the North Sea [33,34], our results showed gradually increasing similarity within the Baltic Sea MPAs with decreasing salinity from west to east. The FB MPA had the lowest mean similarity of 38.8% among the Baltic Sea MPAs as it includes stations from broad habitat types ranging from circa- and infralittoral mixed sediment (hard substrate) to sand and mud [50]. Characteristic species in the FB MPA apart from *A. islandica* included infaunal polychaete species like *Aricidea suecica* and *Levinsenia gracilis,* as well as *Diastylis rathkei, Varicorbula gibba* and *Abra alba*, in line with Schiewer [32], Gogina et al. [34] and Marx et al. [50]. Blue mussel *Mytilus edulis* and infaunal bivalve species like sand gaper *Mya arenaria* were among characteristic taxa for KR MPA, confirming the habitat distribution reported in [50]. Also, in accordance with Marx et al. [50], muddy sediment at RB MPA was dominated by the Baltic tellin *M. balthica*, whereas blue mussels were common in mixed and sand substrate. *Mytilus edulis* as well as *Gammarus salinus* associated with benthic vegetation were typical macrofauna species for AG. At sands of OB, characteristic infaunal bivalve species were *M. arenaria*, *M. balthica* and *Cerastoderma glaucum*; Mytilidae were also common.

The inventory presented here is just a snapshot documenting and comparing macrofauna diversity sampled within two years before the expected official closure for bottom-contact fishing took place. It seemed interesting to check how well our taxa list, which consists of two years of sampling, matches the compilation of the study from Zettler et al. [40], who included long-term databases provided by 11 marine research institutes and private consultancies. We have identified 9 taxa not recorded in the earlier published annotated checklist (marked in yellow in the full species list in Supplementary Material S1). Newly recorded 8 taxa were found in samples from the North Sea MPAs (*Epizoanthus papillosus* (SAR and DGB), *Macropodia tenuirostris* (BRG), *Lepas anatifera* (DGB), *Gilvossius tyrrhenus* (SAR and BRG), *Epimeria cornigera* (DGB), *Malmgrenia lunulata* and *Loimia ramzega* (BRG and DGB) and *Clymenura lankesteri* (DGB)), and one taxon occurred in the MPA Fehmarnbelt (*Alvania punctura*) in the Baltic Sea. *Cylista* sp. (formerly *Sagartia* sp.) was recorded only in the North Sea according to the checklist issued in 2018, whereas we recorded this taxon in our FB samples in 2020 and 2021. Such additional records in new studies were expected and discussed in [40], on the one hand through the introduction of new species and on the other through the spread of marine species from neighboring areas. In their review of non-indigenous species, Lackschewitz et al. [82] reported 159 marine and estuarine taxa, including both macrofauna and macroflora introduced by anthropogenic vectors as well as cryptic species. The number of introduced species detections increased from 9 before, to 48 within, and 65 after the 20th century, partly due to ship traffic, but also due to targeted monitoring programs and growth of taxonomic expertise. The highest number of macrofauna neobiota in the North Sea was represented by bryozoans and tunicates, in the Baltic Sea—by Ponto-Caspian amphipods and mysids. In addition to the actual immigration or even introduction of species, taxonomic revisions are also responsible for the fact that nomenclatures change or species were split up or deleted. One example of such a recently described species is the polychaete *L. ramzega* [83]. Spatial expansion of warm-temperate non-native species into German waters due to water temperature rise or changes in (de-)eutrophication [14,84,85], increasing number of newly introduced species [86], and disappearance of some native taxa are among expected drivers of future changes in species compositions. After closure for bottom-contact fishing in MPAs, those drivers might superimpose on effects of vanished bottom-contact fishing, and will act alongside high natural variability and unpredictable recruitment events particularly relevant in the young and temporally less stable Baltic Sea ecosystem.

### 4.2. Environmental Drivers

Salinity, temperature, sediment parameters describing fractions of sand, mud, gravel and shell, depth and bottom-contact fishing were together responsible for over 68% of the variation in the presence/absence structure of macrofauna data from the North Sea MPAs. The MPA SAR showed the highest taxa number (187 taxa) among North Sea MPAs. The MPA SAR covers 28% of the German EEZ in the North Sea and is characterized by different kinds of sediment parameters [67]. The correlation between sediment parameters and the distribution of macrofauna communities was found in many previous studies [18,40,51,81], although it is often linked to food supply. The distribution of sediments in the German EEZ is heterogeneous, consisting mainly of sand, mud or a mixture of both [87,88]. The mud content correlated, for example, with the abundances of the decapods *Nephrops norvegicus* and *Goneplax rhomboides* [87]. These two species together with high numbers of the holothurian *Paraleptopentacta elongata* were found at stations in the MPA SAR, where the mud content was higher (species list in Supplementary Material S1). MPAs DGB, BRG and AMB are sandbanks and are normally not characterized by a high biodiversity [89]. Due to its geographical position, the DGB MPA had a higher number of taxa than the other two, comprising species typical for the northern North Sea together with species typical for the southern North Sea [20,65]. Typical northern arctic-boreal species were the bivalve *Abra prismatica* and the polychaete *Ophelia limacina* [20]. However, climate change had led to a community shift even on the Doggerbank, and the abundance of northern species decreased at the MPA DGB [20].

Measured near-bottom salinity was by far the strongest factor of changes in (presence/absence-based) community structure on the scale of five studied Baltic Sea MPAs, alone responsible for over 33% of variation (in agreement with multiple studies reporting higher diversity with high salinity ([34,72] and references therein)). Without salinity, mud fraction could explain over 24%. Surprisingly, fishing intensity (subsurface SwAR) was the second most important predictor, explaining 8% of the cumulative effect, followed by modeled long-term averaged temperature (4%), whereas sediment parameters describing fractions of gravel and mud as well as depth each added no more than 2% to the cumulative explained variation. On one hand, this indirect confirmation of trawling impact on macrofaunal biodiversity is in line with recent findings of Bradshaw et al. [90]. This study highlighted for the Swedish part of the southern Baltic Sea that environmental variables (including salinity) affected fauna more than trawling (we will discuss this further in the next section). On the other hand, sediment parameters and depth are commonly considered major environmental forcing factors for macrofauna distribution in the Baltic; therefore, such a small partial effect here, outperformed even by modeled temperature, was somewhat unexpected in the context of previous findings (see e.g., [34]). This discrepancy is likely explained by certain redundancy of variation in those drivers and variation in salinity as the main predictor in this particular dataset, meaning they are capturing similar aspects of the variation in the presence/absence-based community structure (presumably, those covariates would also have more additional explanatory power for abundance- or biomass-based structure).

### 4.3. Bottom-Contact Fishing Intensity

Overall bottom-contact fishing intensity in the considered North Sea MPAs was highest at AMB (Table 3). Bottom-contact fishing seemed to change in ranking between the MPAs over time. Based on the spatial distribution of the surSwAR and subsurSwAR data [62,63], the bottom-contact fishing at the DGB was second highest, followed by the SAR and BRG (Table 3). Even though there is a significant difference between the bottom-contact fishing in the North Sea MPAs, it alone explained over 33.6% (subsurSwAR) of variation in the presence/absence data. In the Baltic Sea, bottom-contact fishing alone could explain 13% of macrofauna variation. It was highest in the Fehmarnbelt MPA (Table 3), followed by Western Rønne Bank (where trawling was mainly active on the muddy northwestern side of the major MPA area) and Odra Bank. It was substantially lower in the Kadetrinne (where shipping traffic is particularly intense) and in the Adler Ground MPA (characterized by riffs avoided by fishers due to gear damage risk). Where bottom-contact fishing occurs, it is often found to be among the most significant disturbances of macrofauna taxonomic [39] and functional composition [91], resulting in clear declines in benthic abundance and species richness [18,92]. Remarkably, despite including the most heavily trawled spots for our Baltic Sea study area, the Fehmarnbelt MPA provided home for the highest number of recorded species (264 taxa) and showed the largest variation in assemblage composition between its stations, not least due to its transition position, variety of habitat types, and highest salinity among Baltic Sea focus areas (see [93]). Generally, in the North and the Baltic Seas, the mobile bottom-contact fishing intensity has more or less decreased since the early 2000s (see Supplementary Material S2 refs. [62,63,64,74]). In the North Sea, the implementation of the Natura 2000 directive seems to be having an effect, but no uniform consensus has yet been implemented to bring fisheries and nature conservation together in a coherent way (see also [94]).

Here we have not studied the influence of other anthropogenic drivers, but among other factors communities are also affected by the construction and exploitation of offshore wind farms (OWF), marine traffic, heavy metal pollution, and changes in oxygen conditions [95]. Large-scale development of OWF has an impact on marine biodiversity due to changes in sediment characteristics and the creation of artificial reefs, the latter causing a doubling of species richness and an increase of abundance by two orders of magnitude. Furthermore, it leads to a decrease or cessation of bottom-contact fishing, prohibited in many OWFs, though fishing avoidance benefits there have yet to be proved [95].

Though temporal variability could not be explicitly analyzed based on datasets considered in our study, it is worth noting that the decline of bottom-contact fishing may have different effects on the macrofauna, depending on how bottom-contact fishing and environmental drivers change and how quickly the communities can adapt. In protected areas where no bottom-contact fishing took place, certain fish species and their macrofauna prey have the opportunity to recover. However, the cumulative effect of decreased direct bottom-contact fishing, changes of the environmental drivers and increased predation pressure may be difficult to disentangle. For macrofauna, especially for epifauna, improved food supply can have a positive impact on population dynamics. Decreased bottom-contact fishing can allow the restoration of mussel banks and seagrass beds.

Some species can adapt quickly to changing environmental conditions (climatic changes), while others are more sensitive [96,97]. The response of the species is driven by the different life histories (growth or age at maturity), differences in morphology (shape and structure) and ecological attributes (like mobility and position on/within the sediment) [42]. The effects of extreme events, for example cold winters in the North Sea can influence habitats for several years, and recovery time of the macrofauna communities from trawling in such disturbed habitats may take just as long [87,98]. When bottom-contact fishing activities change, e.g., in response to sustainable management, changes in community composition and density are expected to follow, but how quick and strong the response will be is dependent on the region. Species that can recover quickly after bottom-contact fishing distribution are decapods like *Crangon crangon*, *Carcinus maenas*, *Corystes cassivelaunus* and *Pagurus bernhardus* [42]. Species with a long lifespan and fragile morphology like bivalves and sessile species (ascidians and bryozoans) showed no short-term recovery after bottom-contact fishing events [42,86,98]. Long-term monitoring is thus crucial to understand the ecosystem changes and to develop appropriate protection measures for benthic macrofauna and their habitats.

To assess the possible impact of bottom-contact fishing on the populations of “key species” [69,70] in the MGF focus areas of the Baltic Sea (*A. islandica* in the FB, *M. balthica* in the RB, and *M. balthica* and *M. arenaria* in the OB), their size-frequency distributions were documented to allow comparison with data planned to be obtained after the fishery exclusion. In FB, the absence of medium size classes (10–30 mm) of *A. islandica* was noticeable, suggesting only occasional mass recruitment success. This lack of cohorts may threaten the continuous development of a stable population, with large, old mussels ensuring its continued existence and dominance. In addition, shell damage from trawling was evaluated [99], suggesting a significant negative impact caused particularly to larger individuals of *A. islandica* by high mechanical forces (traction, pressure) while towing or hauling up the net.

ICES datasets are only of limited use for ecosystem impact studies due to limited spatial resolution. In relation to bottom-contact fishing, these datasets only map the biomass brought ashore, not the biomass (including bycatch) actually removed from the habitat [100,101,102]. For the scale of this study, it currently remains the best available data source, though for small case studies, acoustically derived bottom-contact fishing indices can be the best alternative [103].

Limitations and possible biases of our results should draw attention to differences in methodologies used (overall standardized, but featuring regional details, particularly as in the Baltic in- and epifauna were always sampled together, whereas separate targeting of one or another was more common for the North Sea, as explained in detail in the Materials and Methods section) and the temporal and spatial distribution of stations. The relatively small number of environmental factors used in our statistical analyses, as well as the absence of some other important factors, such as sediment organic matter content or chlorophyll A (that were not available for both studied regions), could have implications for our results, and would likely explain additional variation. Two factors were included for only one of the two regions, namely mean multiannual near-bottom water temperature from the GETM model for the Baltic [73] and the % of shell content > 2000 µm in the sediment for the North Sea. In contrast to the North Sea, where temperature measured at stations during sampling added over 16% to cumulative explained variation, in the Baltic Sea, modelled temperature explained an additional 4% of the cumulative effects on benthic macrofauna structure, whereas measured temperature, though significant, added only 0.7%. As samples in both regions were collected in different seasons and months of the year, these differences most likely reflect variations in thermal regimes and species composition. As for % of shell, it had no significance in the cumulative effect for community composition in the North Sea after considering the effects of salinity, temperature and % of sand fraction.

Syntheses of long-term monitoring data collected over the last two decades under the terms of BfN and other initiatives (already highlighted in the Introduction) should provide a more thorough description and understanding through future research. There is an intense discussion that future data collection, particularly in MPAs, should be revised and developed towards non-invasive sampling. Possibilities here include, for example, the collection of eDNA from water samples (e.g., [104]) or the observation of certain areas using underwater video [72,105]. Even though these methods were confirmed to have great complementary value (e.g., [105]), the taxonomic and quantitative resolution and reliability of data that they can deliver [106] seem, for now, to be insufficient, even for a snapshot assessment like ours. The development of new emerging methods (including the assessment of eDNA persistence and spatial representability, upbuilding of reference libraries and automation of imagery data processing) should go along with their comparison with traditional morphological approaches to support consistency [107,108]. For solid scientific comparison before and after fishing closure, it is still inevitable and essential to use bottom-contacting and dragged scientific equipment (at least in a limited amount) even after the closure. For now, keeping “invasive” scientific gear out of the MPAs may only hamper efficient monitoring. In particular, the importance of long-term ecological research sites in these areas should also be emphasized.

## 5. Conclusions

The baseline inventory of macrobenthic species presented here is important for assessing future faunal changes. Studying biodiversity across German NS and BS MPAs in a collective approach is particularly important for understanding ecological connectivity, integrating conservation strategies, and robustly evaluating the resilience of these ecosystems. A collaborative viewpoint enables the identification of shared species, fostering a more precise understanding of species migration, interactions, and their contributions to overall ecosystem vitality. By adopting a joint perspective, conservation efforts can be strategically enhanced on a broader scale, taking into consideration common threats and species distribution patterns across both regions, thus facilitating more effective planning and management strategies.

## Figures and Tables

**Table 1:** Sampling in 2020–2022 in the North Sea Natura 2000 areas, sampling method (infauna = 0.1 m^2^ van Veen grab, epifauna = ring dredge and beam trawl), number of sampled stations and sampling month. Note: # only four stations had the same locations for the in- and epifauna sampling, but were sampled at different research cruises; * in- and epifauna sampling took place at the same research cruise: at each station, infauna was sampled first, then epifauna.

Study Site	Sampling Methods	No. of Stations	Month
2020			
Sylt Outer Reef (SAR) #	In- and Epifauna	20	May
Borkum Reef Ground (BRG) *	In- and Epifauna	14	July
2021			
Dogger Bank (DGB)	Infauna	20	May
Sylt Outer Reef (SAR)	Infauna	20	May
Dogger Bank (DGB)	Epifauna	25	July
Amrum Bank (AMB)	Epifauna	11	August
2022			
Sylt Outer Reef (SAR)	Epifauna	15	May
Borkum Reef Ground (BRG)	Epifauna	14	July
Amrum Bank (AMB)	Epifauna	11	July

**Table 2:** Number of sampled stations as well as the year and month of sampling of the Baltic Sea MPAs in 2020–2022—always in- and epifauna. * Single stations were visited in other months.

Study Site	No. of Stations in MPA (Close Outside)	Month
2020		
Fehmarnbelt (FB)	29 (12)	June *
Western Rønne Bank (RB)	1	July
Pomeranian Bay with Odra Bank (OB)	4	June–July
Kadetrinne (KR)	6	June
Adler Ground (AG)	8	July
2021		
Fehmarnbelt (FB)	13 (3)	June *
Western Rønne Bank (RB)	6	June
Pomeranian Bay with Odra Bank (OB)	40	June *
Kadetrinne (KR)	11	June
Adler Ground (AG)	14	July–Aug
2022		
Fehmarnbelt (FB)	33 (16)	March, June
Western Rønne Bank (RB)	7 (4)	April, June
Pomeranian Bay with Odra Bank (OB)	40	March, June
Kadetrinne (KR)	4	June
Adler Ground (AG)	6	June

**Table 3:** Overview of the nine MPAs in the North and Baltic Sea, including the total taxa number, the abiotic factors as depth (m), mud (%), gravel (%), temperature (°C), salinity (psu) and oxygen content (mL/L) over the time period 2020–2022, and the bottom-contact fishing intensity (surSwAR and subsurSwAR). SurSwAR and subsurSwAR are averaged per year based on ICES 2016–2020 data [62,63] for the North Sea and 2016–2021 data [64,74] for the Baltic Sea. * At the focus area AMB, only epifauna sampling took place. Values are average per MPA stations ± standard deviation.

Sea	Area	Total Taxa No.	Depth, m	Mud, %	Gravel, %	Temp, °C	Temp Mod, °C	Sal, (psu)	O_2,_ ML/L	Trawling	
										sur SwAR	Subsurswar
North Sea	SAR	187	43 ± 1.2	16.2 ± 8.3	2.6 ± 9.9	7.5 ± 0.4	/	34.3 ± 0.2	/	0.61 ± 0.62	0.40 ± 0.32
	BRG	135	30 ± 1.2	0.5 ± 0.5	1.2 ± 2.2	16.4 ± 0.6	/	33.4 ± 0.2	/	0.2 ± 0.22	0.03 ± 0.02
	DGB	143	43.3 ± 0.6	0.2 ± 0.1	0	11 ± 0.6	/	34.5 ± 0.03	/	1.08 ± 0.2	0.68 ± 0.3
	AMB *	50	11.4 ± 2.7	/	/	18.2 ± 0.2	/	30.9 ± 0.6	/	3.04 ± 0.96	1.58 ± 0.5
Baltic Sea	FB	264	22 ± 5	33.5 ± 23.5	2.8 ± 8.2	8.1 ± 4	7.9 ± 0	19.3 ± 2.9	6 ± 1.8	2.64 ± 2.45	0.21 ± 0.19
	RB	58	34 ± 5	15 ± 15.8	5.8 ± 14.3	6.9 ± 2.2	5.7 ± 0.1	10.7 ± 1.6	6 ± 0.7	0.85 ± 1.05	0.07 ± 0.08
	OB	56	14 ± 2	0.3 ± 0.4	0 ± 0	13.1 ± 4.4	8.4 ±0.1	8.2 ± 1.6	6.9 ± 1.3	0.88 ± 0.67	0.07 ± 0.05
	KR	141	19 ± 4	26.6 ± 33.8	2.8 ± 7.6	10.6 ± 1	7.4 ± 0	16.5 ± 2.4	5.2 ± 0.9	0.21 ± 0.22	0.01 ± 0.01
	AG	62	16 ± 7	0 ± 0	3 ± 9.5	12.9 ± 3.4	7.6 ± 0.9	7.8 ± 0.7	6.6 ± 0.5	0.03 ± 0.04	0.00 ± 0.00

**Table 4:** Results of the SIMPER analysis: ten characteristic taxa contributing most to the average similarity within the MPAs in the North Sea.

SAR		BRG		DGB	
Mean similarity: 62.3%	*Abra alba*	Mean similarity: 57.5%	*Aonides paucibranchiata*	Mean similarity: 67.5%	*Amphiura filiformis*
	*Amphiura filiformis*		*Astropecten irregularis*		Anthozoa
	*Astropecten irregularis*		*Ensis* spp.		*Aora gracilis*
	*Chamelea striatula*		*Lanice conchilega*		*Asterias rubens*
	*Corystes cassivelaunus*		*Liocarcinus holsatus*		*Astropecten irregularis*
	*Cylichna cylindracea*		*Spio symphyta*		*Bathyporeia elegans*
	*Echinocardium cordatum*		*Spiophanes bombyx*		*Bathyporeia guilliamsoniana*
	*Eudorella truncatula*		*Thia scutellata*		*Dosinia lupinus*
	*Hyala vitrea*		*Asterias rubens*		*Echinocyamus pusillus*
	*Kurtiella bidentata*		*Bathyporeia guilliamsoniana*		*Euspira nitida*

**Table 5:** Results of the SIMPER analysis: ten characteristic taxa contributing most to the average similarity within the MPAs in the Baltic Sea.

FB		KR		RB		AG		OB	
Mean similarity: 38.8%	*Aricidea suecica*	Mean similarity: 46.9%	*Mytilus edulis*	Mean similarity: 49.2%	*Macoma balthica*	Mean similarity: 60.2%	*Mytilus edulis*	Mean similarity: 64.0%	*Peringia ulvae*
	*Scoloplos armiger*		*Peringia ulvae*		*Peringia ulvae*		*Peringia ulvae*		*Pygospio elegans*
	*Ophiura albida*		*Bylgides sarsi*		*Scoloplos armiger*		*Gammarus salinus*		Tubificinae
	*Varicorbula gibba*		*Pygospio elegans*		*Diastylis rathkei*		*Einhornia crustulenta*		*Mytilus edulis*
	*Diastylis rathkei*		*Eucratea loricata*		*Pontoporeia femorata*		*Pygospio elegans*		*Mya arenaria*
	*Kurtiella bidentata*		*Kurtiella bidentata*		*Halicryptus spinulosus*		*Hediste diversicolor*		*Hediste diversicolor*
	Tubificinae		*Asterias rubens*		*Bylgides sarsi*		Tubificinae		*Marenzelleria viridis*
	*Levinsenia gracilis*		*Mya arenaria*		*Hediste diversicolor*		*Jaera albifrons*		*Macoma balthica*
	*Abra alba*		*Nephtys caeca*		*Capitella capitata*		*Amphibalanus improvisus*		*Cerastoderma glaucum*
	*Paradoneis eliasoni*		*Diastylis rathkei*		*Mya arenaria*		*Bylgides sarsi*		*Streblospio shrubsolii*

**Table 6:** Number of Taxa with a critical Red List status in the focus areas of the North Sea and Baltic Sea (source: [78,79]).

Status	Both	North Sea	Baltic Sea
Near Threatened	16	7	9
Extremely Rare	26	12	16
Threat of Unknown Extent	47	28	30
Threatened	10	6	5
Highly Threatened	8	5	5
Threatened with Extinction	3	1	2

## Data Availability

The original contributions presented in the study are included in the article/Supplementary Material, further inquiries can be directed to the corresponding author/s. The LEGRA infauna data from the Baltic Sea are also going to be available via an online portal of the Federal Agency for Nature Conservation at the end of 2024 (contact: kathrin.heinicke@bfn.de), and the MGF data are going to be available via an online portal, PANGEA, by the end of 2024 (contact: mayya.gogina@io-warnemuende.de). CTD data from the DGB of the MGF-North Sea project benthos and macrofauna team by Senckenberg are available at https://doi.pangaea.de/10.1594/PANGAEA.964365 (accessed on 22 March 2024).

## Supplementary Materials

The following supporting information can be downloaded at: https://www.mdpi.com/article/10.3390/biology13060389/s1, Supplementary Materials S1: Joint species list for the German MPAs in the North and Baltic Seas in 2020–2022. Temporarily resolved list for the Baltic Sea. Results of DistLM. Supplementary Materials S2: Temporal dynamics of trawling activity. Biodiversity values expressed as mean Shannon–Wiener indices.
