# Supplementary material for: Baseline Inventory of Benthic Macrofauna in German Marine Protected Areas (2020–2022) before Closure for Bottom-Contact Fishing
Source: Biology (Basel). 2024 May 28;13(6):389. doi: 10.3390/biology13060389 (PMC11201066; doi:10.3390/biology13060389)
Supplement: Supplementary file 1 [file biology-13-00389-s001.zip › Supplementary Files/Supplementary_Material_S2_revised.docx]

**Baseline Inventory of Benthic Macrofauna in German Marine Protected Areas (2020 - 2022) Before Closure for Bottom-Contact Fishing**

Mayya Gogina, Sarah Joy Hahn, Ramona Ohde, Angelika Brandt, Stefan Forster, Ingrid Kröncke, Martin Powilleit, Katharina Romoth, Moritz Sonnewald, Michael L. Zettler

**Supplementary Material S2**

**Temporal dynamics of trawling activity**

A B


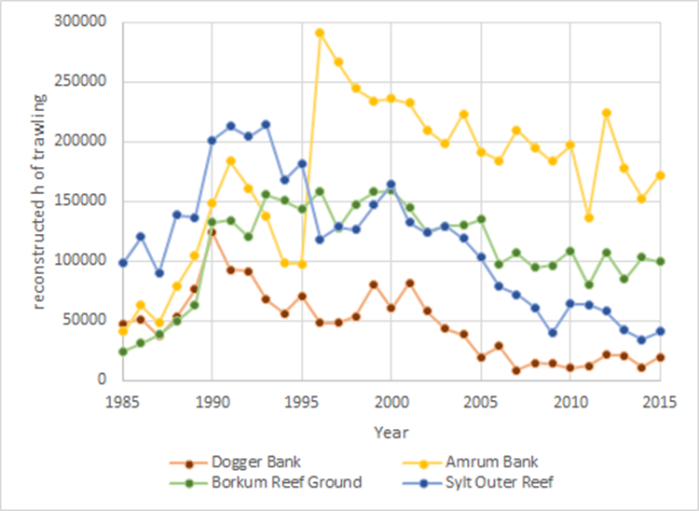
 **
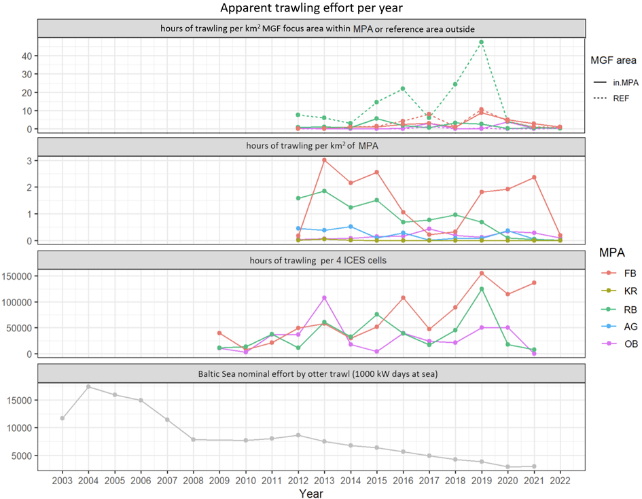
**

**Figure S2.1.** A: Reconstructed trawling effort in the four study sites of the North Sea in ICES transects 40F3, 40F4, 39F3, 39F4 (Dogger Bank); 38F7, 38F8, 37F7, 37F8 (Amrum Bank); 37F5, 37F6, 36F5, 36F6 (Borkum Reef Ground); 39F6, 39F7, 38F6 and 38F7 (Sylt Outer Reef) from 1985 to 2015. Data are from the Couce et al. [110] dataset. B: Reconstructed apparent trawling effort in the Baltic Sea: in MGF focus areas and in all 5 MPA within German EEZ standartised per km^2^ area (two upper panes, data source:<https://globalfishingwatch.org> [111], accessed on 19 September 2023), total annual hours of trawling in the four ICES c-square (each covering an area of 0.05 x 0.05 degree corresponding to approx. 18 km^2^) surrounding the MGF focus areas (based on available ICES data from 2009-2016), as well as annual nominal otter trawl efforts for the entire Baltic Sea region (ICES [81]).

Trawling activity North Sea

For the analysis of the ground contact fishery in the corresponding study areas, each of the four surrounding ICES rectangles were identified. The data set of Couce et al. [110] allowed to record the reconstructed hours of trawling, both beam- and otter-trawling per year in the corresponding ICES rectangles from 1985 to 2015. This shows that trawling intensity changes over time in all areas. All sites show an increase in trawling effort between 1985 and 1990, measured in reconstructed hours per year. While the Sylt Outer Reef had the highest trawling effort from 1985 to 1995, a strong decrease can be seen since 1993. In contrast, the trawling effort on the Amrum Bank rises sharply from 1995 onwards. Compared to the other three areas, Dogger Bank had the comparatively lowest trawling effort. From 2001 at the latest, a decline in trawling effort can be recognized in the ICES rectangles of all the areas studied.

Trawling activity Baltic Sea

The reconstructed apparent trawling effort in the Baltic Sea is presented in Fig. S2.1 B. In order to translate the nominal fishing effort (thousand kW days at sea, available for 2003-2016) to fishing effort (kW fishing hours, available for 2016-2021) published by ICES for the Baltic Sea ecoregion, here a factor 11.5 was used, that was derived based on 2016 values present in both sets.

***Biodiversity***

A B


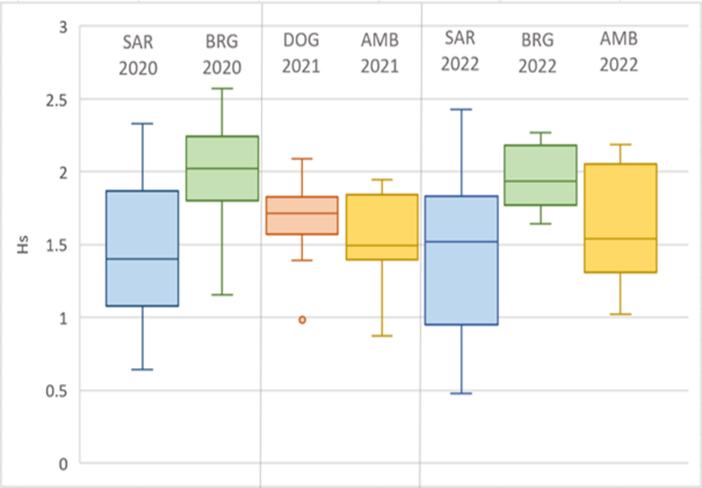

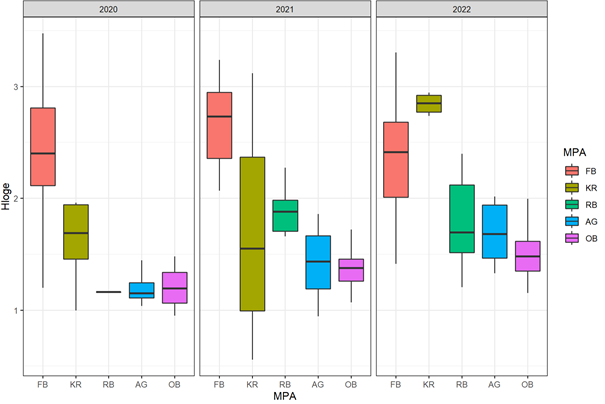


**Figure S2.2.** Variability of Shannon-Wiener diversity of the (A) 4 study sites Sylt Outer Reef (blue), Borkum Reef Ground (green), Dogger Bank (orange) and Amrum Bank (yellow) in the years 2020 – 2022 based on epifauna data; (B) mean Shannon-Wiener indices of the Baltic Sea study sites in the years 2020 – 2022.
